# An Efficient Management System for Wireless Sensor Networks

**DOI:** 10.3390/s101211400

**Published:** 2010-12-13

**Authors:** Yi-Wei Ma, Jiann-Liang Chen, Yueh-Min Huang, Mei-Yu Lee

**Affiliations:** 1 Department of Engineering Science, National Cheng Kung University, Tainan, Taiwan; E-Mail: n9897106@mail.ncku.edu.tw; 2 Department of Electrical Engineering, National Taiwan University of Science and Technology, Taipei, Taiwan; E-Mail: lchen@mail.ntust.edu.tw; 3 Department of Applied Geoinformatics, Chia Nan University of Pharmacy and Science, Tainan, Taiwan; 4 Department of Computer Science & Information Engineering, National Dong Hwa University Hualien, Taiwan; E-Mail: m9221509@ems.ndhu.edu.tw

**Keywords:** wireless sensor network, management system, fault management, performance management, configuration management, SNMP

## Abstract

Wireless sensor networks have garnered considerable attention recently. Networks typically have many sensor nodes, and are used in commercial, medical, scientific, and military applications for sensing and monitoring the physical world. Many researchers have attempted to improve wireless sensor network management efficiency. A Simple Network Management Protocol (SNMP)-based sensor network management system was developed that is a convenient and effective way for managers to monitor and control sensor network operations. This paper proposes a novel *WSNManagement* system that can show the connections stated of relationships among sensor nodes and can be used for monitoring, collecting, and analyzing information obtained by wireless sensor networks. The proposed network management system uses collected information for system configuration. The function of performance analysis facilitates convenient management of sensors. Experimental results show that the proposed method enhances the alive rate of an overall sensor node system, reduces the packet lost rate by roughly 5%, and reduces delay time by roughly 0.2 seconds. Performance analysis demonstrates that the proposed system is effective for wireless sensor network management.

## 1. Introduction

Wireless sensor networks have garnered considerable attention recently. The ability of sensor networks to sense, communicate and process data has led to the rapid development of such networks. Many researchers have attempted to improve wireless sensor network management efficiency. Wireless sensors networks can sense and monitor information from the physical world, and are used in the scientific, medical, and commercial domains. Sensor nodes can operate autonomously to collect and exchange information in a particular environment [1]. Wireless sensor networks with large nodes will acquire very large amounts of data. Network management then becomes very difficult. Supporting convenient and effective network management is crucial in wireless sensor networks. This paper intends to improved network configuration, performance, and fault management using *WSNManagement*, a novel management system for wireless sensor networks [2,3].

The Simple Network Management Protocol (SNMP) provides good management capability for TCP/IP-based networks and it is the most widely used network management tool. SNMP is an application layer protocol that promotes the exchange of information between network devices. It allows network administrators to manage network performance, identify network problems, and enhance network performance. SNMP is widely supported by the vendor community, and has been successfully used to manage both wired and wireless networks. We utilized the SNMP-based functions to manage wireless sensor networks.

The remainder of this paper is organized as follows. Section 2 reviews work on sensor management problems. Section 3 describes the proposed *WSNManagement* system in detail. Section 4 presents the simulation environment, and implementation and analysis results. Section 5 presents the performance of the *WSNManagement* system. Conclusions are given in Section 6.

## 2. Related Works

### 2.1. Simple Network Management Protocol (SNMP)

**Figure 1.**
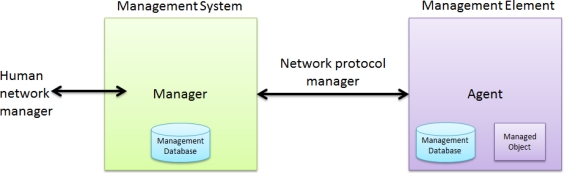
SNMP-based Management Model.

SNMP is based on the manager and agent model, which consists of a manager, agent, and management information database [4]. The manager model provides an interface between a human manager and a management system. The agent model provides an interface between the manager and the physical device being managed (Figure 1). The manager and agent use management information and a relatively small set of commands to exchange information [5].

### 2.2. Wireless Sensor Network

Wireless sensor networks combine the capabilities of low-power processors and wireless communication modules for particular services. Cheap, portable, low-power, tiny multifunctional sensor nodes have recently been developed for wireless communication and digital electronic devices [6].

The two most important tasks for a sensor node are information collection and transferring data traffic. Information gathering encompasses environment sensing and information processing. Traffic transmission may transfer original sensing data or forward traffic as in an intermediate relay in a multi-hop path. Sensor nodes can be either distributed as a mass or one by one in a sensor field. Once deployed, sensor nodes start working and may auto-organize, wait for a command to work, or start sensing and forwarding data. Sensor nodes usually relay a data stream to a base station or command node (management node) based on a regular rule or event.

Sensor nodes in wireless sensor networks are spread over a region and communicate using point-to-point wireless communication. Sensor nodes collect, process, and send data obtained from an environment. Wireless sensor networks can use three different nodes.

**Figure 2.**
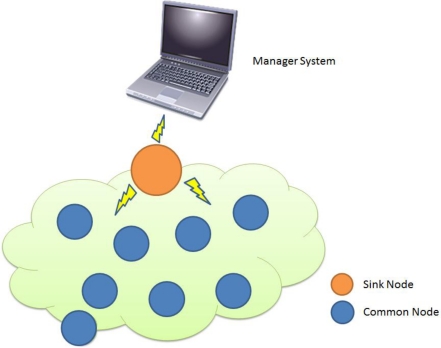
Sensor Network Architecture.

- Common node: This node collects environment information and then sends data to a sink node.
- Sink node: This node receives data from sensor nodes and may store, process, or compress the data.
- Manager node: This function displays data for manager analysis. It also allows a manager to query sensor nodes. A manager node is connected to a sink node directly via a wireless connection or through another communication channel. Figure 2 shows these manager nodes and the communication method.

### 2.3. Sensor Network Management Functions

The objective of wireless sensor networks is to monitor and control a physical environment and achieve specific goals. Wireless sensor networks are designed to promote productivity when combined with the functions of an organizational of configuration, operation, management, and maintenance services in sensor networks [7].

A MANNA (Management Architecture for Wireless Sensor Network) architecture was proposed in Reference [8]. The functions of a wireless sensor network differ from those of a traditional network. In the MANNA architecture, the fault, security, performance, and accounting functions are dependent on configuration management.

Configuration management is a high correlation of sensor nodes in wireless sensor network management system. The goal of *WSNManagement* is to monitor sensor network collection, processing, and transmission of data, and ultimately control an environment. Problems or unexpected situations may adversely affect the configuration of services provided. Functions of management have been identified configuration of network-level requirements, which is the network operating environment, monitoring the environment, and node deployment.

As failures can occur in a wireless sensor network, wireless sensor network management differs from traditional network management. Sensor failure can be due to energy shortages, connection interruptions, and environmental changes. Thus, a sensor network must be robust, and must function even when individual nodes, networks, or even services fail. In addition to problems caused by energy issues, other problems can occur in wireless sensor networks, such as those associated with communications, services, data processing, physical equipment failure, operator violation, or security non-safety.

Providing wireless sensor network security features is difficult because of their ad hoc features, intermittent connections, and wireless communication and resource constraints. Furthermore, wireless sensor networks are subject to internal, external, accidental, and malicious security threats. Information or resources can be modified, stolen, deleted, or lost, and service can be interrupted. Even when a wireless sensor network is secure, the network environment can be unsafe or vulnerable [9–12].

The two main objectives for performance management of a WSN are acquisition of quality information and distribution of services. A trade-off exists in performance management among the highest number of managed parameters, the highest energy consumption, and lowest network lifetime. Conversely, when parameter values are not obtained, managing a network appropriately is impossible.

## 3. *WSNManagement* Architecture

Wireless sensor networks are applied in many fields. As network type and structure are versatile in complex network environments, a network manager must be able to control and understand information and the operating situation to ensure that network resources are used efficiently. However, these goals are not achievable when done by a human. Therefore, a network manager must work with network management systems to integrate monitoring tasks and control network traffic. An effective network management system must provide the following functions to assist network managers.

(1) A network management system must automatically search and display network ability and its connection on the Graphical User Interface (GUI). The system must also provide a network operator with a thorough understanding of the network environment, such that the operator can monitor and control remote devices.

(2) A network management system must collect data and assess network utilization, and make adjustments according to analytical results.

(3) When an abnormal network situation occurs, the system must automatically notify management staff, identify the probable cause, and provide a solution.

The structure and functionality of the *WSNManagement* network management system is presented in this section. How a network manager manages a sensor network with the *WSNManagement* system is also described.

### 3.1. WSNManagement *System Architecture*

The network manager can use an easy operation environment to monitor and examine a network system. The *WSNManagement* system can manage all abilities on a sensor, and control communication, configuration, and backup support for a network management system. These are practical issues and crucial to network management. Furthermore, diagnosing situations by statistical analysis of different network applications and forecasting trends are important to future network adjustments.

**Figure 3.**
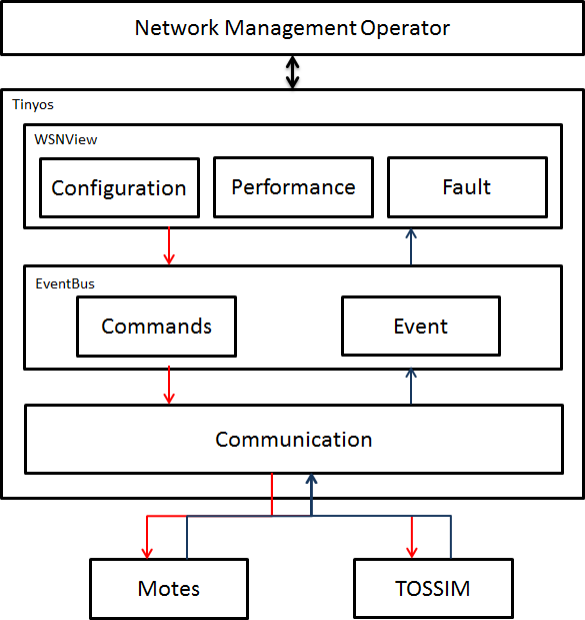
*WSNManagement* System Architecture.

Network administrators use the Configuration, Performance, and Fault functions of the *WSNManagement* user interface to obtain values from a sensor node through the Communication technology and TOSSIM [12] or mote according to Commands and Events provided by the Event-Bus. The Command signal is sent by the *WSNManagement* user interface through the TOSSIM as an administrator command. Figure 3 shows the *WSNManagement* system architecture.

### 3.2. WSNManagement *Management*

- Configuration Management
  Configuration management searches and sets sensor status in a sensor network to control and monitor the status of sensor nodes. The sink node is linked to each sensor node through the Communication function, which can determine network status. Network administrators can manage sensor networks via the GUI (e.g., redeploying, increasing, or decreasing the sensor node based on sensor node density). Configuration management is used to obtain data from sensor networks.
- Performance Management
  The purpose of performance and administration control is to keep a system operating, and to ensure secure data transmission. Performance and administration control can help an administrator monitor sensors, collect performance data, record detected values (e.g., temperature and luminosity) from sensors, and monitor power consumption. Via these data, an administrator can analyze and predict the development of an entire network, such that the administrator can solve problems or upgrade a system as soon as network performance declines.
- Fault Management
  Function of fault administrator control enhances network reliability. For network awareness, one must monitor network status. By analyzing historical data, consecutive events, or event queries from sensors, the fault administrator control process can obtain current data. Fault administrator control can be used to identify errors associated with sensor hardware faults, power shortages, lost connections, and environment changes.

## 4. *WSNManagement* System Implementation

**Figure 4.**
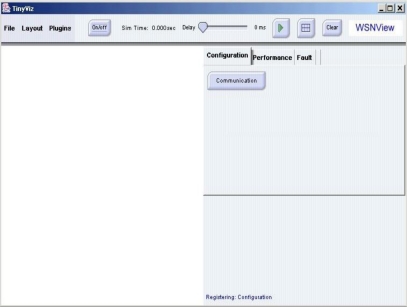
*WSNManagement* User Interface.

The *WSNManagement* system is implemented with Java as a plug-in of TinyViz and connects to Tinyos [14] motes and TOSSIM through the TCP port. Figure 4 shows the user interface of the *WSNManagement* system. The *WSNManagement* system has a list of functions at the top of the window. A node display window is on the left and network management function selection tabs are on the right under the functions list.

The “file” function loads the location map of sensor nodes. The “layout” function can change the layout of sensor nodes to a grid or a randomly generated one. The “plugin” function is used to add user-defined plugins to the system. The “play” button is on the function list and represented by a triangular green arrow. When the “play” button is pressed, the simulation starts and the “play” button changes into a “pause” button (two vertical grey bars). Sliding the “delay” slide (center of the function list) slows the rate at which events are generated and processed from the simulation. The “grid” button (a grey grid) displays grid lines in the node display window. The “clear” button clears simulation information and displays sensor nodes.

They are generated randomly or deployed according to an input map in the locations of mote nodes. When simulation is in random mode, the *WSNManagement* system places motes in random locations in the node display window. Users can move any node and change its location in the entire sensing field; this triggers an event and changes the “real” location of motes in the TOSSIM. The wireless sensor network manager can perform management tasks and obtain useful information using the configuration, performance, and fault tabs in the network management tab. These special features include deployment, sensing coverage, system life and faulty sensor nodes. The sensor network management functions are described in detail in following sections.

### 4.1. Configuration Management

**Figure 5.**
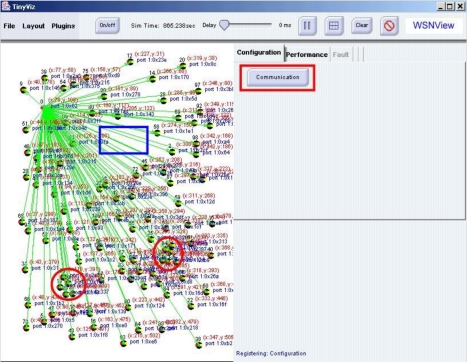
Configuration of Sensor Deployment.

Sensor nodes in the experiments were deployed randomly. The sensor with the id 0 was chosen as the sink node. The sink node communicated with each node via broadcasting and collected data from each sensor node. Figure 5 shows the sensor node display window.

**Figure 6.**
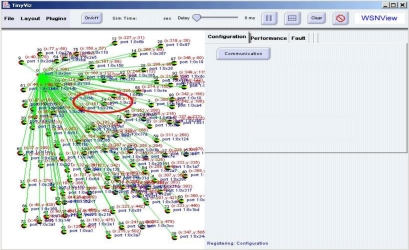
Configuration after Adjusting the Sensor Deployment.

By examining the sensor node display window, a network manager can analyze the deployment of sensor nodes. Additionally, the network manager can read the coordinates of sensors in each sensor node. Furthermore, the network manager can determine that no sensor node exists in the rectangular area and that the density of nodes in the circular area is excessive. In the simulation case, the network manager can adjust the locations of sensor nodes by dragging and grouping nodes to meet the requirement of the sensor network system. Figure 6 shows the new configuration after adjustments. In the case of mote nodes deployment, the network manager can remove mote nodes in the circular area and redeploy them in the rectangular area to increase distribution uniformity.

**Figure 7.**
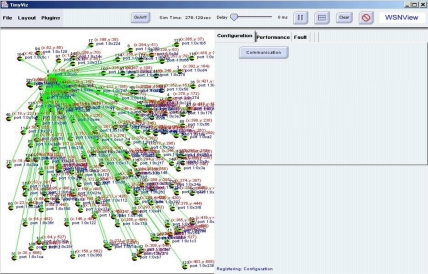
Configuration after Redeployment.

When moving sensor nodes to a dangerous area is impossible, the network manager can deploy sensor nodes to areas that need additional sensors to meet system requirements. Figure 7 shows the outcome of altering sensor deployment in the sensor network. Hence, the network manager can manage the sensor network configuration.

### 4.2. Performance Management

**Figure 8.**
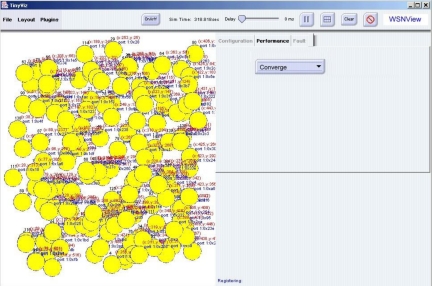
Sensing Coverage Display.

Sensing coverage is an important metric of sensor network performance. During performance management, the performance tab displays sensing coverage of the current designated sensor in the sensor node in the *WSNManagement* system. Figure 8 shows the sensing coverage of the current sensor node deployment. We assume the range of each sensor node is 10 units. In a real case, each sensor node can have multiple sensors. After deployment, a network manager can examine the configuration to determine coverage.

The other performance metric is prolonged system life. Because sensor nodes are battery powered, power is a limited resource for all sensor networks. By examining sensing coverage and identifying the areas in which sensor node coverage overlaps, network managers can deactivate redundant sensor nodes temporarily until other nodes run out of power.

### 4.3. Fault Management

**Figure 9.**
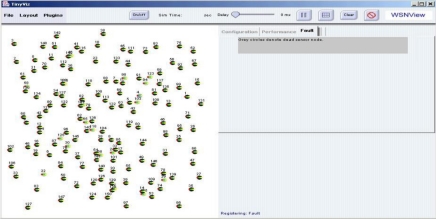
Display after Sensor Nodes Run Out of their Power.

Sensor nodes are easily broken, communication can be obstructed by buildings and trees, and sensor node power is limited. Dead sensor nodes, faults, must be monitored by network managers. When performing fault management, a network manager can select the fault tab, which displays dead sensor nodes in grey in the *WSNManagement* system. Figure 9 shows faulty sensor nodes in the current deployment. The network manager can assess the effect of these faulty nodes on the entire system and decide whether to deploy new nodes to keep the system functioning properly.

**Figure 10.**
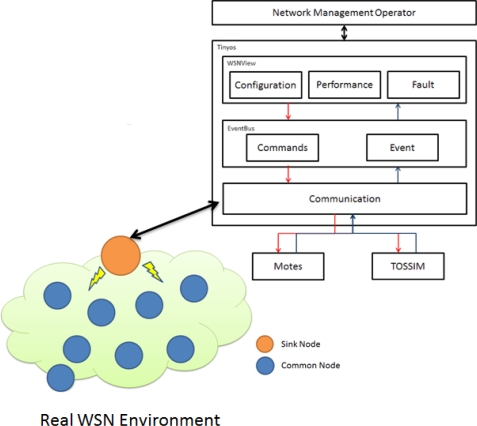
Integrated Real Environment and *WSNmanagement* system.

This work of *WSNManagement* system provided Communication, Event bus, and WSNView module, can be applied to the real networks to enhance management efficiency. This contributes a novel and original researches. In our proposed system, real wireless sensor network can be combined to obtain actual node information from the outside environment. Figure 10 shows the integrated *WSNmanagement* system.

## 5. Performance Analysis

This work proposed the *WSNManagement* system for performance analysis. Using this system, one can determine whether to use the configuration, performance, or fault management mechanisms for performance comparisons. The simulation environment was built for assessing the alive rate for nodes, the packet loss rate, and delay time for performance comparisons. The detailed parameter settings are as follows. The simulation environment contains a sink node and common node. The common node can identify environmental changes in temperature and humidity, and transmit these data back to the user. The simulation environment is close to reflect the real network environment to get performance result. The term with *WSNmanagement* means the environment using WSN management and the term without *WSNmanagement* means the environment not using WSN management.

- Alive means sensor nodes in the sensor network are alive.
- The packet loss rate is the number of packets lost sent from the source minus the number of packets received at the destination.
- The delay time is the average time required to send a packet from a source to the receiver.

**Figure 11.**
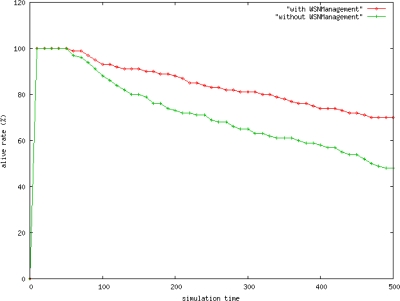
System Alive Rate.

Figure 11 shows the node alive rate of the network. One can use the *WSNManagement* mechanism to enhance the number of alive nodes in a network.

**Figure 12.**
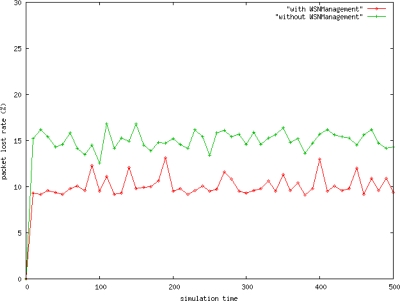
System Packet Loss Rate.

Figure 12 shows that the packet loss rate for data transmission with the *WSNManagement* mechanism is about 5% less than the one without *WSNManagement* mechanism. Simulation results demonstrate that the algorithm effectively enhances network data transmission services. These experimental results further indicate that transmission through the control network node can improve network traffic stability, reduce the packet loss rate, and increase the data arrival rate.

**Figure 13.**
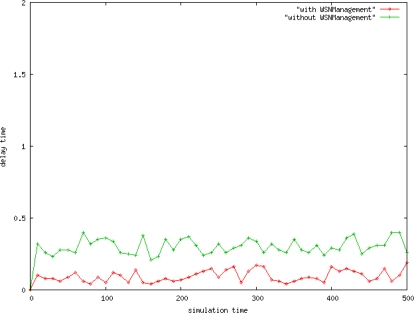
System Delay Time.

The *WSNManagement* system has a shorter delay, which is 0.2 seconds shorter than sensor networks without the *WSNManagement* system (Figure 13). The performance of the proposed framework for selecting the shortest network routing for *WSNManagement* data transfer is better than that without the *WSNManagement* system.

## 6. Conclusions

This work proposed a novel system that can show connection stated in wireless sensor networks relationships among sensor nodes and can monitor, collect, and analyze information from a wireless sensor network. This work of *WSNManagement* system provided Communication, Event bus, and WSNView module, can be applied to the real networks to enhance management efficiency. This contributes a novel and original researches. In our proposed system, real wireless sensor network can be combined to obtain actual node information from the outside environment. For the performance analysis demonstrates that the proposed enhances the alive rate, reduces the packet lost rate and delay time. The system interface and sensor network were examined in detail using the popular sensor network TinyOS mote and its simulation environment TOSSIM. The events and commands of TinyOS were described and categorized by the *WSNManagement* system. The special features of sensor network management, including deployment, sensing coverage, system life and faulty sensor nodes, were extracted and implemented in the *WSNManagement* system. The wireless sensor network management functions covered configuration, performance, and fault management for various situations.
